# Towards an Elastographic Atlas of Brain Anatomy

**DOI:** 10.1371/journal.pone.0071807

**Published:** 2013-08-14

**Authors:** Jing Guo, Sebastian Hirsch, Andreas Fehlner, Sebastian Papazoglou, Michael Scheel, Juergen Braun, Ingolf Sack

**Affiliations:** 1 Department of Radiology, Charité – Universitätsmedizin Berlin, Campus Charité Mitte, Berlin, Germany; 2 Institute of Medical Informatics, Charité, Universitätsmedizin Berlin, Campus Benjamin Franklin, Berlin, Germany; Rensselaer Polytechnic Institute, United States of America

## Abstract

Cerebral viscoelastic constants can be measured in a noninvasive, image-based way by magnetic resonance elastography (MRE) for the detection of neurological disorders. However, MRE brain maps of viscoelastic constants are still limited by low spatial resolution. Here we introduce three-dimensional multifrequency MRE of the brain combined with a novel reconstruction algorithm based on a model-free multifrequency inversion for calculating spatially resolved viscoelastic parameter maps of the human brain corresponding to the dynamic range of shear oscillations between 30 and 60 Hz. Maps of two viscoelastic parameters, the magnitude and the phase angle of the complex shear modulus, |G*| and *φ*, were obtained and normalized to group templates of 23 healthy volunteers in the age range of 22 to 72 years. This atlas of the anatomy of brain mechanics reveals a significant contrast in the stiffness parameter |G*| between different anatomical regions such as white matter (WM; 1.252±0.260 kPa), the corpus callosum genu (CCG; 1.104±0.280 kPa), the thalamus (TH; 1.058±0.208 kPa) and the head of the caudate nucleus (HCN; 0.649±0.101 kPa). *φ*, which is sensitive to the lossy behavior of the tissue, was in the order of CCG (1.011±0.172), TH (1.037±0.173), CN (0.906±0.257) and WM (0.854±0.169). The proposed method provides the first normalized maps of brain viscoelasticity with anatomical details in subcortical regions and provides useful background data for clinical applications of cerebral MRE.

## Introduction

Magnetic resonance imaging (MRI) is inarguably one of the most powerful neuroradiological modalities. In daily routine clinical use, MRI based on the relaxivity of water protons is invaluable for the localization and characterization of pathologies such as tumors, hemorrhage, flow obstruction, or inflammation. Furthermore, MRI methods sensitive to network structures, such as diffusion tensor imaging (DTI), have significantly contributed to our current knowledge of the brain's structures and anatomy [1]. Like the majority of MRI-based methods, neuroradiological MRI depicts brain morphology based on the amount of water protons present and their relaxivity or mobility.

Alternatively, anatomy can also be defined by the constitutive properties of tissues based on the firmness and the mechanical interconnectedness of the underlying tissue matrix. With this approach, physical parameters such as shear elasticity or shear viscosity provide a key to the understanding of multiscalar mechanical structures such as viscoelastic networks consisting of cells and elements of the extracellular matrix.

MR elastography (MRE) can measure the shear modulus of cerebral tissue, providing an imaging parameter that is highly sensitive to the microarchitecture of the tissue [2]. In principle, the capabilities of MRE can be used to delineate anatomical regions of the brain, based on their shear elastic properties [3], [4], [5]. However, since the shear modulus is recovered from the tissue's response to externally induced shear waves, MRE is regularly limited by artifacts related to the solution of the inverse problem in time-harmonic elastography [6].

For this reason, maps of the viscoelastic parameters of the brain in vivo, with a spatial resolution comparable to that of standard MRI have not yet been presented. As a diffuse method that measures the 'global' shear modulus of the whole brain, cerebral MRE has been reported to be sensitive to physiological aging [7], to changes related to diseases such as multiple sclerosis [8], Alzheimer's disease [9], and normal pressure hydrocephalus [10] in humans, and to demyelination [11] and inflammation [12] in mouse models. Preliminary conclusions regarding regional variations in the viscoelastic parameters of the living brain have recently emerged [13], [14], [15], [16], [17].

We hypothesize that the diagnostic value of cerebral MRE would be tremendously increased by overcoming the current limits of resolution, and by providing an atlas of the viscoelastic anatomy of the healthy human brain, as a body of reference data. To achieve these goals, in the present study we used for the first time three-dimensional multifrequency MRE (3DMMRE) and multifrequency dual elasto-visco (MDEV) parameter reconstruction in the brain. 3DMMRE with MDEV inversion was recently introduced for high-resolution mechanical imaging of the liver and spleen [18]. MDEV parameters including the magnitude and the phase of the complex shear modulus are independently obtained from an overdetermined set of wave equations in a fast and numerically stable procedure. The number of wave equations, and hence the stability of the inversion procedure, increases by adding experimental data such as wave field data at different vibration frequencies to MDEV inversion. This approach relies on averaging wave patterns and their Laplacians over frequency, prior to the inversion (as will be further elaborated in the Theory part). The validity of this approach has been demonstrated with both phantom and in vivo data [18]. Recent advances in cerebral MRE provide methods for fast acquisition of wave fields in the brain with high resolution (8 mm^3^ voxel size) [14], [19], [20], enabling the repetition of 3D MRE at multiple drive frequencies within short acquisition times.

In this study, we use the full information acquired by 3DMMRE for the generation of normalized maps of viscoelasticity of the brain obtained in 23 healthy volunteers. We aim to use this information to create a reference atlas of the distribution of viscoelasticities in the human brain. We note that this proposed mechanical atlas corresponds to a dynamic range between 30 and 60 Hz oscillation frequency, which is the common shear wave window of cerebral MRE [13], [14], [15], [16], [17], and may thus be of general interest as background data on brain viscoelasticity.

In the next section we will briefly review the essential equations for understanding MDEV inversion. In the Methods section we will present the technical aspects of MDEV inversion as applied to brain data, and introduce the details necessary to understand the normalization procedure and the analysis of the regional variation of viscoelastic parameters. The results are discussed taking into account the technical aspects and viscoelastic networks in the brain.

## Theory

### The Complex Shear Modulus

The vector field of externally induced waves 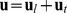
measured by MRE is composed of longitudinal (compression) waves 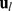 and transverse (shear) waves 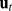. Helmholtz decomposition of **u** yields the curl field **c**, which is free of compression terms, i.e.,
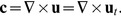
(1)both **u** and **c** can be represented by complex quantities (denoted in the following by an asterisk), corresponding to the harmonic vibration field after Fourier decomposition, 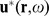 and 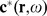 with position vector **r** and angular drive frequency *ω*.

The governing equation of the complex curl field 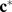 is the Helmholtz equation,
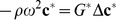
(2)which is a set of independent scalar equations where Δ denotes either the 2D- or 3D-Laplacian applied component-wise to the curl field **c** [21]. *ρ* is the material density and 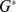 is the isotropic complex shear modulus. Eq.(2) relies on assumptions such as linear viscoelasticity, isotropy, and local homogeneity [22] which are commonly made in MRE. Classically, 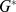 is represented by its real and imaginary parts (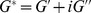), which are synonymous with the storage modulus 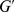 and the loss modulus 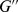, respectively. In our inversion approach, another representation of 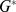 is used:



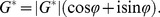
(3)The magnitude of the complex shear modulus 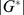 quantifies the amount of storage and loss properties – both are expected to rise as the network density increases in biological tissue. The loss angle, 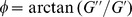 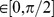, is sensitive to the geometrical properties of the mechanical lattice, as has been demonstrated in several studies [23], [24], [25], [26].

### Multifrequency Dual Elasto-visco (MDEV) Parameter Recovery

We rewrite eq.(2) according to the representation of 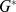 by 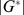 and 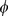 [27]:
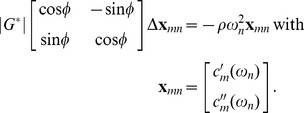
(4)with the indices *m* and *n* corresponding to the *m*-th component and *n*-th drive frequency of the curl field. As can be seen from eq.(4), the Laplace operator causes a scaled rotation of the vector **x** with angle 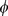. Thus, the scalar product of 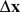 and 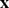,
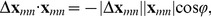
(5)can be solved for 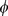 in a least-squares sense by 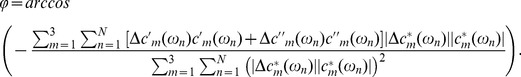
(6)Having analyzed the rotation of 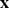 resulting from the Laplace operator, we now address its change in length. Therefore, it is sufficient to solve the magnitude of the Helmholtz equation (eq.(2)),
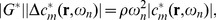
(7)in a similar way as given by eq.(6):
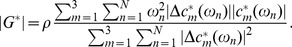
(8)


## Methods

### Ethics Statement

The study was approved by the Ethics Committee of the Charité - Universitätsmedizin Berlin and informed written consent was obtained from all volunteers.

### Subjects

3DMMRE was used in 23 healthy volunteers with no history of neurological abnormality (11 women; age range, 22 to 72 years; mean age ± standard deviation (SD), 41±18 years).

### Imaging Sequence and Wave Excitation

**Figure 1:**
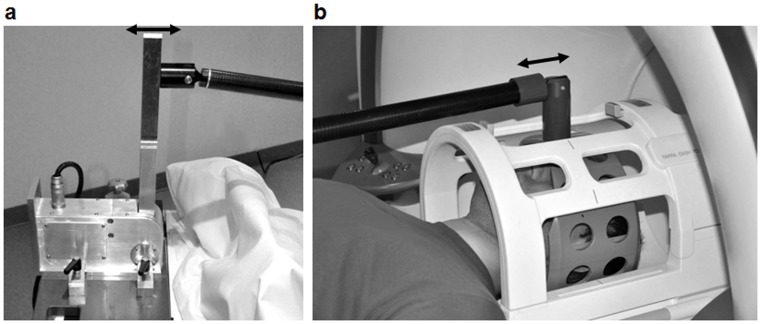
Experimental setup of MRE of the brain: a) Nonmagnetic driver placed at the end of the patient table. b) Passive actuator integrated in the head coil and connected to a) by a carbon fiber piston. The main vibration direction is indicated by black arrows.

All experiments were conducted on a 1.5-T MRI scanner (Magnetom Sonata; Siemens Erlangen, Germany) using a quadrature head coil. A single-shot spin-echo echoplanar imaging sequence with a trapezoidal flow-compensated motion-encoding gradient (MEG) was applied along all three encoding directions for motion field acquisition [18]. Harmonic motion was induced in the brain by a cradle placed beneath the volunteer's head [3] and connected to a nonmagnetic vibration generator at the end of the patient table. The driver was constructed based on piezoceramics [28] capable of generating horizontal vibration amplitudes on the order of 0.2 mm, which were amplified twentyfold by a vertical lever. As a result, the main vibration direction was along the feet-head direction and was adjusted with an adapted amplifier to a 1 mm maximum amplitude, which was well tolerated by all volunteers. A diagram of the experimental setup is shown in figure 1; the subject in this figure has given written informed consent, as outlined in the PLOS consent form, to publication of this photograph.

**Figure 2:**
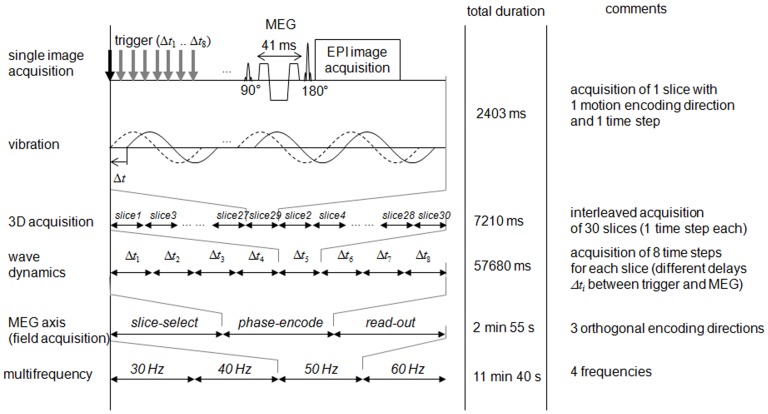
Sequence timing diagram for acquisition of wave fields (three Cartesian motion components) in 30 slices at eight time steps (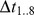) during a vibration period 1/*f*.

Stimulation was initiated by the scanner 100 ms prior to the start of the MEG to allow for the time the waves propagate from the actuator into the head and to minimize transient effects. Four vibration frequencies of 30, 40, 50, and 60 Hz were consecutively applied. For each drive frequency, 30 adjacent transverse image slices of 2×2×2 mm^3^ resolution were recorded in all three motion-encoding directions, and at eight instances over a vibration period. A total of 720 images were acquired in 2 min and 55 seconds, resulting in a total acquisition time of approximately 12 min. Further imaging parameters are: repetition time (TR), 7210 ms; echo time (TE), 99 ms; field of view (FoV), 176 × 192 mm^2^; matrix size 88 × 96; MEG frequencies were matched with the vibration frequencies; MEG amplitude, 30 mT/m. A scheme of the imaging sequence is shown in figure 2.

### Data Processing

**Figure 3:**
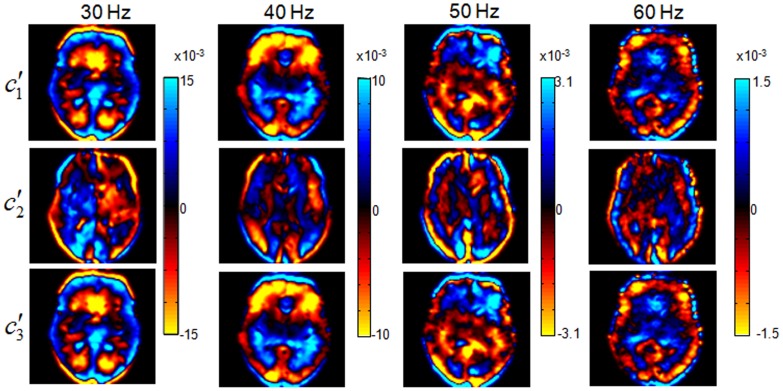
Preprocessed wave fields used for MDEV inversion in a single slice. Shown are the curl components 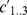
(real parts of 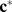 after Fourier transformation).

Raw phase data were i) unwrapped using Flynn's algorithm [29], ii) smoothed by a 3D Gaussian filter with a cubic kernel of 3 pixel edge length, iii) scaled to the dimension of radians per meter by the factor given in eq.(2) of [30], and iv) Fourier-transformed along the time axis (eight instances over one vibration cycle). The curl components 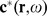 were calculated from the complex harmonic field 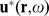 at drive frequency *ω* and low-pass filtered for noise reduction applying a 2D-butterworth kernel with a threshold of 100 m^−1^. Finally, the filtered curl components (Fig. 3) were used in the inversion of eqs.(6) and (8) to calculate 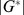 and 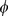, assuming a density of the brain of 1.0 g/cm^3^.

### Normalization and Registration

A template was created based on the 3D-MRE magnitude data sets of all 23 volunteers using Advanced Normalization Tools (ANTS) [31]. Then, the 23 individual 3D MRE data sets were registered and transformed to the template space using their own deformation map and the affine matrix created during the generation of the template. Cross correlation was applied as the similarity metric, and the symmetric normalization (SyN) transformation model was used for diffeomorphic image registration.

The template built from the 23 subject data sets was then registered to the standard MNI152lin brain 2 mm T1 atlas from the FMRIB software library (FSL) database [32] using the warp command from ANTS. The affine transformation matrix obtained by the registration procedure was applied to each normalized image for warping it into the fixed template space of the MNI152 T1 atlas resulting in 30 usable transverse image slices.

The viscoelastic parameter maps of each subject were transformed into the standard atlas domain by deformations prescribed by the transformation of MRE magnitude images into the standard T1 atlas space as mentioned above. Finally, elastographic atlases were created by averaging normalized maps of all 23 subjects.

### Segmentation

We evaluated tissue viscoelasticity in four regions of interest (ROI's) obtained either by tissue probability map based segmentation with additional thresholding (white matter; WM) or manual segmentation (thalamus, head of the caudate nucleus, corpus callosum genu; TH, HCN, CCG, respectively). Cortical gray matter was not analyzed in this study since the thickness of this tissue is on the order of, or even below the image resolution, thereby imposing inconsistencies due to registration bias. Furthermore, cortical areas are susceptible to boundary artifacts by the inversion. Segmentation was performed on MNI152lin_brain 2 mm T1 atlas and the resulting ROI masks were applied to 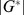 and 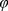 maps for spatial averaging of the viscoelastic parameters.

A two-tailed t-test with a 5% significance level was applied to the regional comparisons of 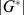 and 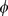.

## Results

### The Effect of Noise on MDEV Parameter Recovery

**Figure 4:**
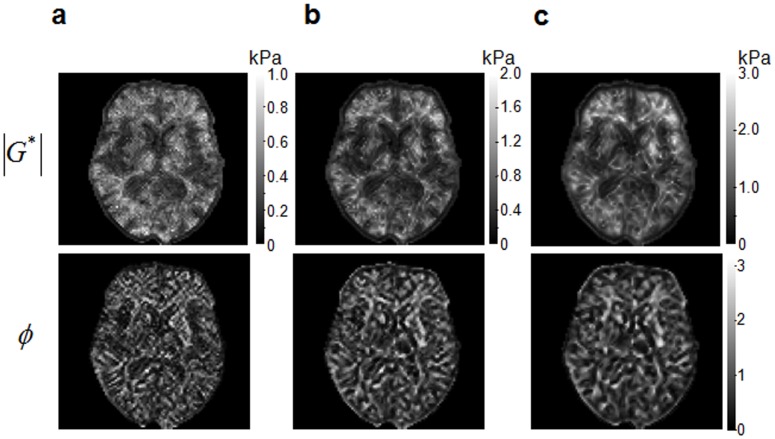
Changes in MDEV maps resulting from increasing noise suppression in a single transverse slice. 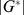
 and *φ* are reconstructed from: (a) unsmoothed and unfiltered wave data; (b) smoothed wave data prior to curl calculations; (c) smoothed wave data prior to curl calculations and subsequent noise filter (wave number limit of 100 m^−1^. The processing steps used for (c) were applied to the rest of the paper.

Figure 4 presents 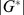 and 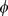 maps in one central slice of a healthy volunteer with increasing noise suppression. Column (a) presents 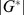 and 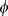 without any filter, i.e. the wave data were used without further preprocessing for calculating the curl components 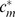, which were then used without modification in inversion eqs.(6) and (8). Maps in column (b) were obtained based on curl components 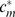 calculated using preprocessed (smoothed) wave data, as described in the Methods section. However, different from our final procedure, here we applied no further filtering after the calculation of 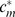. Column (c) represents the results obtained by the processing procedure described in the Methods section, i.e. smoothing of the wave data prior to curl calculation, combined with noise suppression in 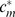. Upon request, we will share this set of 3DMMRE data to interested researchers in the field.

**Figure 5:**
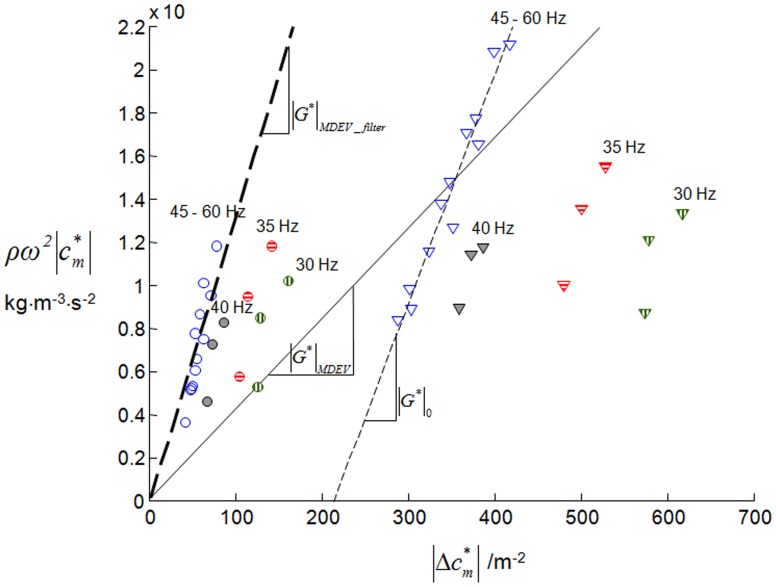
The influence of noise on MDEV inversion given by eq.(8). The right-hand side and the left-hand side of eq.(7) are calculated from experimental data at individual drive frequencies from 30 Hz to 60 Hz (note, for demonstration purposes, experiments were performed in a volunteer with 7 excitation frequencies ranging from 30 to 60 Hz in increments of 5 Hz). The triangles correspond to unfiltered curl components, and the circles are obtained by applying the filter described in the Methods section (see also fig. 4c). Data from different frequencies are color-coded with different filling patterns (open symbols: 45–60 Hz; solid gray: 40 Hz; horizontal line pattern: 35 Hz; vertical line pattern: 30 Hz). The slopes of the fit lines correspond to modulus 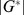. According to eq.(8), the fit lines are forced to run through the origin, resulting in severe underestimation of 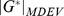. A better implementation of least-squares inversion would account for an offset as done by the fit yielding 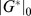 (here only the consistent high-frequency data [blue symbols] are considered). The noise-related offset is suppressed by an appropriate noise filter as used in figure 4c yielding 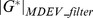.

As indicated by the limits of the vertical color bars, the spatially averaged 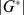
values increase from (a) to (c) with 0.360, 0.589 and 0.910 kPa, respectively. Compared to the range of elasticity values of brain parenchyma obtained by other MRE methods [3], [14], MDEV inversion tends to underestimate 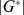 with less noise treatment. Using the proposed filtering procedure, 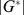 is in the range of values expected from static indentation experiments on brain tissue slices [33], but lower than previous data of 2DMMRE [16]. It is known that Helmholtz inversion of noisy data tends to result in underestimation of the modulus [34]. Similarly, the effect of noise on our MDEV inversion is analyzed in figure 5, where we show the spatially averaged right-hand side of eq.(7) plotted against the averaged Laplacian 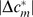. In this representation, each data point defines a 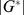 value which is the slope of a line running through that point and the origin. This representation is identical to the inversion of eq.(7) and can be extended to multiple data points and a least-squares fit according to eq.(8). In figure 5 we used multiple frequencies, i.e. 30, 35, to 60 Hz. For each frequency we found three 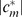 components, which were averaged over the whole brain parenchyma visible in a central slice of a volunteer. MDEV inversion combines in 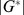 all curl components at each frequency to the slope of a linear least-squares fit running through zero. This is demonstrated by the closed line graph for the curl components of 45–60 Hz, yielding 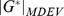
<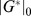. 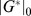 is the magnitude shear modulus obtained by a linear fit excluding zero (dashed thin line). The resulting positive intercept on the abscissa is due to noise, since noise affects 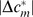 more than 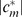. Thus, noise reduction by low-pass filtering reduces this offset, as is demonstrated by data points treated by the filters we used for MDEV inversion and described in the Methods section (circle symbols). The resulting slope of the linear fit 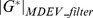 agrees well with 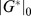. It is notable that data points corresponding to low frequencies (<45 Hz) display slopes clearly different from the high frequency data (≥ 45 Hz). We attribute this effect to viscosity, which causes a strong dispersion of the complex shear modulus of brain tissue within the used frequency range [7]. Therefore, incorporating low-frequency data (<45 Hz) into the linear fit would diminish the slope 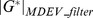 giving lower shear modulus values in this frequency range.

The sensitivity of *φ* to noise was analyzed in a similar manner. The averaged *φ* values presented in fig. 4 decrease from (b) to (c) by 0.057, showing that the right-hand side of eq.(5) (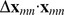) is less prone to noise than the **x**-term on the left-hand side (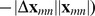, yielding an underestimation of 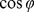, and finally - unlike 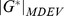 - an overestimation of *φ*.

### Elastographic Atlases

**Figure 6:**
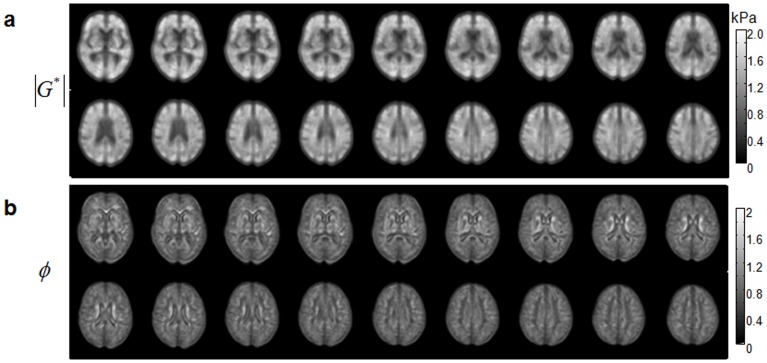
Elastographic atlases according to 3DMMRE at 30, 40, 50 and 60 Hz vibration frequencies and MDEV inversion of the data of 23 healthy volunteers.

Figure 6 presents 18 normalized transverse slices (out of 30) of 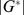
and *φ* averaged over 23 healthy volunteers in the frequency range from 30 Hz to 60 Hz.

**Figure 7:**
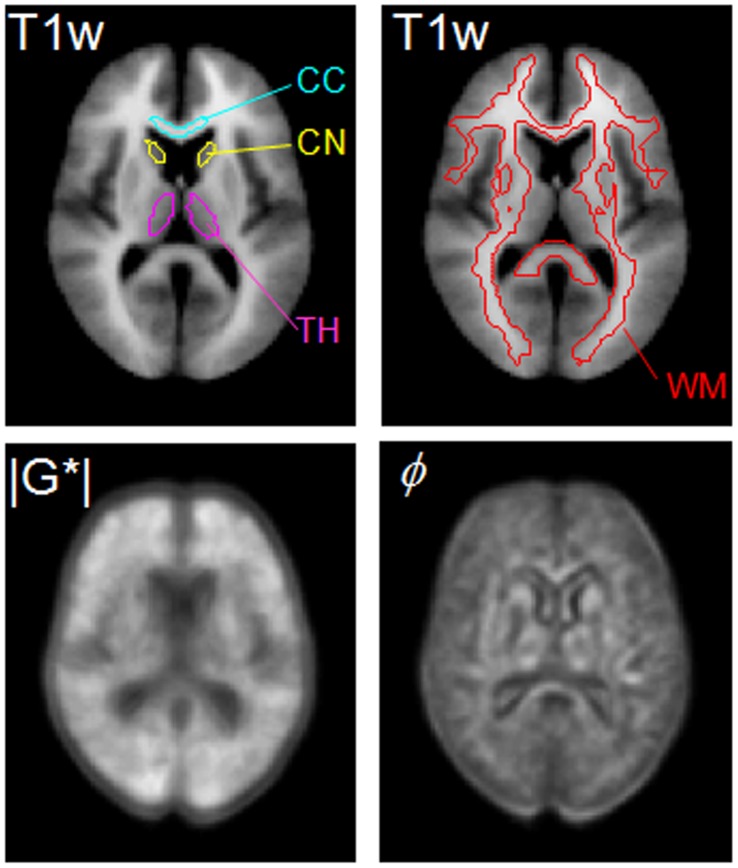
For illustration, one central slice of the T1-weighted template data (T1w), 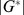
maps and *φ* maps are selectively shown with all four anatomical regions, i.e., the head of caudate nucleus (HCN), thalamus (TH), corpus callosum genu (CCG), and white matter (WM). Regions of HCN, TH, and CCG were manually selected while the WM region was automatically segmented from the T1w.

In figure 7, a central slice of both viscoelastic parameters taken from figure 6 is magnified for comparison with the corresponding T1-weighted template slice, and for illustration of the regions of interest. The ROIs shown in figure 7 were used for spatial averaging, and the averaged viscoelastic constants are collected in Table 1(a). The corresponding standard deviation (SD) provides an indication of the heterogeneity of 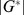 and *φ* within the investigated area.

**Table 1:** Spatially averaged MRE parameters in four regions of the brain.

	a		b	
	Normalized (atlas)		Non-normalized (subject map)	
	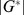 /kPa	*φ*	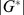 /kPa	*φ*
WM	1.252 (0.260)	0.854 (0.169)	1.269 (0.110)	0.868 (0.061)
TH	1.058 (0.208)	1.037 (0.173)	1.080 (0.186)	1.055 (0.201)
HCN	0.649 (0.101)	0.906 (0.257)	0.626 (0.152)	0.921 (0.300)
CCG	1.104 (0.280)	1.011 (0.172)	1.131 (0.191)	1.031 (0.233)

(a) Spatially averaged MRE parameters 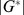 and *φ* in four regions of the brain (WM – white mater, TH – thalamus, HCN – head of caudate nucleus, CCG – corpus callosum genu) based on normalized atlases averaged over 23 healthy volunteers (fig.6) in the drive frequency range from 30 Hz to 60 Hz, standard deviations [SD] given in brackets represent regional variation. (b) Mean values of the spatially averaged 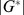 and *φ* over the same ROIs in the non-normalized 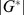 and *φ* maps of each volunteer, standard deviations [SD] given in brackets represent subject variation among 23 volunteers.

**Figure 8:**
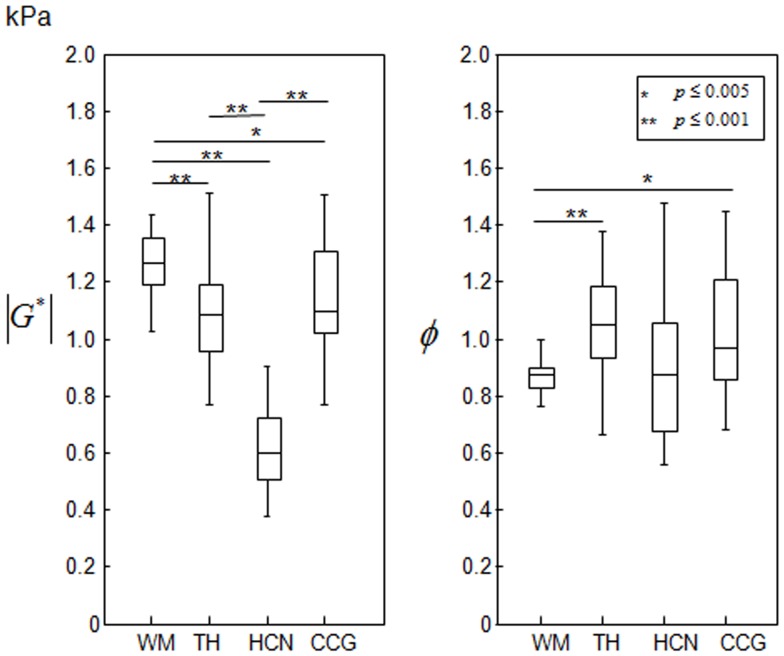
Regional differences estimated using non-normalized elastographic maps of subjects obtained in the range of drive frequencies from 30 Hz to 60 Hz. The boxplot depicts the lower and upper quartiles and the 50^th^ percentile (median) from the 23 subject group. The full data range is presented by whiskers.

For validation, we additionally analyzed the 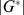 and *φ* values within the same anatomical regions of the original (non-normalized) elastograms. The group mean values of the four anatomical regions are plotted in figure 8 and tabulated in Table 1(b). Their standard deviations refer to the inter-subject variations of 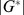 and *φ*. Comparing Table 1(a) and 1(b) we see that the results from non-normalized maps agree well with the values displayed in the atlas. However, Table 1(b) also shows that our magnitude shear modulus 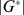
is in the range of 0.6 kPa to 1.3 kPa, which is lower than expected from previous MRE studies [4], [7], [14] but is within the range of values reported from indentation experiments, rheometry, and in vitro MRE [33], [35]. Figure 8 and Table 1(b) also indicate inter-regional mechanical differences. Upon combining data of non-normalized maps from all volunteers, we observed decreasing 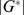-values in the order of WM>CCG>TH>HCN, which was significant for all regions with P<0.005, except for CCG>TH.

Figure 8b analyzes the regional variation of the loss angle *φ,* which has not yet been exploited in the literature of cerebral MRE. MDEV reconstruction yielded *φ* values between 0.854 and 1.011 in the order of WM<HCN<CCG<TH (see Table 1(a)).

## Discussion

The generation of elastographic maps of in vivo human brain is an active research field. In this study we introduce several innovations as steps toward high-resolution cerebral MRE in a clinical experimental setting: i) we used a nonmagnetic driver system that has never before been tested for brain MRE. The actuator placed at the end of the patient table was simple to connect to the head cradle, and sustained an effective wave excitation within the common frequency band of human brain MRE. As such, the proposed driver provided a system-independent MRI hardware supplement suitable for cerebral multifrequency MRE in clinical practice. ii) We applied for the first time 3DMMRE to the brain and iii) combined this method with MDEV inversion. The first atlases of 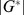 and *φ* of the human brain generated in this manner reveal the mechanical properties of the brain parenchyma in unprecedented detail. Standard Helmholtz analysis of cerebral wave fields in the common frequency range between 25 and 62 Hz reports storage moduli between 1.64 and 2.58 kHz [16], which is consistent with the results of cerebral MRE reported by others [14], [17].

The elastic moduli we measured in this study are approximately half of those values. We attribute this discrepancy to the fact that MDEV inversion possibly over-weights low frequencies, which naturally have higher amplitudes. It has been demonstrated in phantoms that MDEV inversion is capable of reproducibly yielding correct elasticity values [18], [36]. However, phantoms usually display less viscous damping than the living brain, and thus better mimic the situation of constant parameters as implied in our inversion. The frequency dispersion of 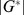 and *φ* in brain tissue is averaged by MDEV inversion in order to treat wave nodes and amplitude nulls occurring at single frequencies, thereby improving the resolution of the resulting parameter maps. Clearly, this improved resolution is achieved at the expense of comparability with published values. As 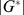 and *φ* have not yet been reported for brain tissue, further studies are needed for their validation. Nevertheless, considering the wide range of elasticity values yielded by other methods [5], our novel mechanical contrast may contribute to the ongoing search for valid mechanical parameters in the brain.

Importantly, 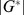 and *φ* are model-free. Comparison of *φ* to the viscoelastic powerlaw constant 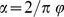 used in previous 2DMMRE studies would assume the validity of the springpot model, which implies a constant 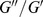
ratio [26]. As a consequence, the predictability of *φ* by *α* is limited, and *φ* values based on *α* may differ from the values determined by fitting 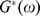 dispersion functions in 2DMMRE [7], [8], [10], [37]. Nonetheless, in homogeneous materials, the 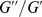
ratio is sensitive to the geometry of viscoelastic networks as has recently been demonstrated by [24], where viscoelastic structure elements were added to agarose-based MRE phantoms, resulting in transformation of initially elastic and nonviscous materials into lossy materials with high network density. This observation corroborates previous findings in irregular polymer networks demonstrating that *φ* increases with the amount of unlinked and dangling chains [23]. However, other MRE-related factors such as shear wave scattering at cortical sulci and tissue interfaces [36] can influence the attenuation of shear waves and may thus render *φ* particularly prone to overestimation.

Gray-matter (GM) tissue, consisting of somata, dendrites, and axons, is less organized and lacks network support compared to WM fiber bundles of aligned myelinated axons. In earlier studies, higher elasticity values were found for WM than for GM, using 3DMRE and 2DMMRE [13], [14], [16] as well as ex vivo indentation experiments [33].

As seen from figure 8, HCN has the lowest stiffness of all analyzed regions; this is probably because it contains mainly clusters of neuronal cell bodies. The tissue architecture of the thalamus is more complex; the internal medullary lamina, a thin sheet of white matter, runs longitudinally through the thalamus and separates the thalamus into subregions with different nuclear groups. Consisting of a mixture of WM and GM structures, the thalamus has 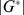 between that of WM and HCN. The CCG is primarily composed of densely packed fibers. Our results show relatively high values of 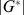 and *φ* in CCG, however, without accounting for the directionality of viscoelastic constants in anisotropic media. As callosal fibers are largely parallel to the transverse imaging plane in the CCG, the imaging plane is not normal to the fiber axis, and would not be a plane of symmetry for this transversely isotropic material. As has recently been shown [38], shear moduli along the cortical spinal fiber tracks (C_13_ and C_23_) are significantly softer than C_12_, which is related to the plane of isotropy in this orthotropic frame. For this reason we may have measured lower 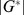
values in the CCG than in WM; however, conclusions about specific anisotropic elasticity constants require knowledge of the directions of polarization and propagation of the waves [39], which is undefined in our setup. Regardless, anisotropy remains to be addressed in future work on mechanical atlases of the brain.

In general, an adequate evaluation of the complexity and heterogeneity of brain tissue urgently requires further improvements in the spatial resolution of viscoelastic parameter maps, taking into account anisotropy [38] and poroelastic properties [14], [19], [40]. Isotropic MRE parameter recovery with an 8 mm^3^ cubic voxel resolution [14], [19], [20] is a preliminary step towards high-resolution atlases of the mechanical constitution of in vivo brain. Our study is further limited by a small group size, combined genders and ages, and a limited transverse size of the investigated region of 6 cm. Further improvements of viscoelastic parameter maps is warranted, and can be accomplished by including more drive frequencies, and using 3T MRI scanners for noise reduction.

In summary, combining a nonmagnetic shear wave source, fast 3DMMRE, and MDEV reconstruction allowed us to produce for the first time a frequency-averaged elastographic atlas of the central cerebrum, with spatially resolved subregions including the WM, TH, HCN and the CCG. Viscoelastic properties were mapped by magnitude and phase of the complex shear modulus, providing information about the rigidity and density of different types of mechanical networks in brain tissue. The viscoelasticity maps presented here will provide background data for clinical cerebral MRE, and, analogous to standard MRI-based brain atlases, will enable the registration and segmentation of tissue structures based on the mechanical contrast in MRE.
